# Advances and Challenges in Developing Efficient Graphene Oxide-Based ZnO Photocatalysts for Dye Photo-Oxidation

**DOI:** 10.3390/nano10050932

**Published:** 2020-05-12

**Authors:** Asim Ali Yaqoob, Nur Habibah binti Mohd Noor, Albert Serrà, Mohamad Nasir Mohamad Ibrahim

**Affiliations:** 1 School of Chemical Sciences, Universiti Sains Malaysia, 11800 Penang, Malaysia; asim.yaqoob@student.usm.my (A.A.Y.); nurhabibah970717@gmail.com (N.H.b.M.N.); 2 Empa, Swiss Federal Laboratories for Materials Science and Technology, Laboratory for Mechanics of Materials and Nanostructures, Feuerwerkerstrasse 39, CH-3602 Thun, Switzerland

**Keywords:** photocatalysis, dye photodegradation, graphene oxide, zinc oxide, wastewater treatment

## Abstract

The efficient remediation of organic dyes from wastewater is increasingly valuable in water treatment technology, largely owing to the tons of hazardous chemicals currently and constantly released into rivers and seas from various industries, including the paper, pharmaceutical, textile, and dye production industries. Using solar energy as an inexhaustible source, photocatalysis ranks among the most promising wastewater treatment techniques for eliminating persistent organic pollutants and new emerging contaminants. In that context, developing efficient photocatalysts using sunlight irradiation and effectively integrating them into reactors, however, pose major challenges in the technologically relevant application of photocatalysts. As a potential solution, graphene oxide (GO)-based zinc oxide (ZnO) nanocomposites may be used together with different components (i.e., ZnO and GO-based materials) to overcome the drawbacks of ZnO photocatalysts. Indeed, mounting evidence suggests that using GO-based ZnO nanocomposites can promote light absorption, charge separation, charge transportation, and photo-oxidation of dyes. Despite such advances, viable, low-cost GO-based ZnO nanocomposite photocatalysts with sufficient efficiency, stability, and photostability remain to be developed, especially ones that can be integrated into photocatalytic reactors. This article offers a concise overview of state-of-the-art GO-based ZnO nanocomposites and the principal challenges in developing them.

## 1. Introduction

Rapid industrialization as well as population growth in the past 50 years have made actions against water scarcity and water pollution urgent priorities for governments, industries, and civil society worldwide. The 2020 United Nations World Water Development Report estimated that nearly 748 million people are unaware of the shortage of pure water drinking and that the water required by manufacturing industries will increase by a staggering 400% by 2050 [1]. Each year, approximately 3.2 million people die, especially in developing countries, because they cannot access clean water or sufficiently sanitary environments [2]. Beyond that, several types of inorganic and organic compounds consumed in large amounts have become a dangerous factor in the devastation of the world’s ecology.

Against that background, more efficient processes for decontaminating water urgently need to be developed as conventional wastewater treatments become inefficient for remediating a range of persistent organic pollutants (e.g., pesticides, herbicides, dyes, and surfactants) and new emerging contaminants (e.g., trace organic compounds, nanoparticles, microplastics, cyanotoxins, and antibiotics). Significant efforts have been invested in improving natural aerobic methods, the separation of membranes, coagulation, precipitation, flocculation, Fenton reactions, photocatalysis, and adsorption. However, several limitations—the complexity and the time-consuming nature of the processes, the higher cost of operations and chemicals used, aggregate sludge production, and difficulties with separation—inhibit their potential use [3]. Against of all those limitations, advanced oxidation processes, based upon the catalytic or photocatalytic heterogeneous or homogeneous oxidation of complex organic pollutants are considered to rank among the best candidate technologies for efficient wastewater treatment. Meanwhile, among advanced oxidation processes, photocatalysis is possibly the most promising due to the simple, environmentally friendly conditions required, including the use of solar light as an energy source and, in turn, lower energy costs and less energy consumption. Heterogeneous photocatalysis could be efficient for mineralizing organic pollutants (i.e., total oxidation) and photo reducing toxic inorganic heavy metals such as hexavalent chromium [4,5]. However, various scientific and technical barriers prevent the use of heterogeneous photocatalysts—among others, their inefficient integration into fixed reactors or the limited post- recovery of unfixed photocatalysts after water treatment; the wide bandgap of potential photocatalysts, which restricts their use only under UV irradiation and, in turn, significantly increases costs, namely because solar light contains only 5% of UV light; and low durability and efficiency of photocatalytic treatment processes [6,7,8].

Since 1972, various metal oxides have been investigated as heterogeneous photocatalysts for wastewater treatment; examples include zinc oxide (ZnO), iron (III) oxide (${\mathrm{Fe}}_{2}O_{3}$), titanium dioxide (${\mathrm{TiO}}_{2}$), tungsten trioxide (${\mathrm{WO}}_{3}$), zirconia (${\mathrm{ZrO}}_{2}$), niobium pentoxide (${\mathrm{Nb}}_{2}O_{5}$), and vanadium oxide ($V_{2}O_{5}$) [9,10]. Among those semiconductors, ZnO has shown great potential in recent years due to its unique characteristics, including chemical stability, biocompatibility, a robust ability amid oxidation, acceptable photocatalytic performance, exceptional photosensitivity, good pyroelectric and piezoelectric properties due to its shape and size, and potentially simple synthesis using various scalable methods [11]. ZnO-nanostructured materials are smaller than bulk materials and have a high specific surface area (i.e., high active sites), and both of those qualities facilitate the adsorption of pollutants and subsequent mineralization when irradiated with UV light due to the production of photogenerated charge carriers and reactive oxygen species (ROS) such as hydroxyl radicals. However, the relatively broad bandgap—approximately 3.37 eV—requires the use of UV light, the rapid recombination of electron-hole pairs, and low photocorrosion resistance, thus inhibiting their practical use. To date, several strategies have been used to improve the performance of ZnO, including ion doping, composite, metal loading, coupling it with semiconductors, structural design improvements, and combining it with carbon materials, all in a bid to achieve photocatalysts driven by visible light or to extend the lifetime of photo-generated electron-hole pairs [12]. Even so, one of the most promising strategies to that end has been combining ZnO with carbon-based materials such as graphene and its derivatives. Such materials exhibit a remarkable pore structure, as well as high electrical, thermal, and chemical properties and adsorptive capacity. Among carbon-based materials, graphene oxide (GO) is an ideal candidate for integration with ZnO-based photocatalysts, due to not only its outstanding dispersive ability inside any solvent thanks to the surface functionality of carboxyl and hydroxyl groups but also to its potential to enable charge separation when conjugated with ZnO [13]. Recently, the use of GO-based ZnO nanomaterials for photocatalytic applications has become more popular due to their excellent physicochemical and photo-electrochemical properties, which make them more competitive than other nanomaterials. The structural features of GO-based nanomaterials can significantly improve the photocatalytic performance of ZnO thanks to their π- conjugation structure that exhibits excellent electronic mobility, which both promotes the separation of electron-hole pairs on the ZnO surface and improves the harvesting of light energy in the visible region. In addition, their high specific surface, with a large number of active sites and excellent support properties, allows the formation of efficient heterojunctions with intimal contact between ZnO and GO, which accelerates the charge transfer and catalyst dispersion, both of which improve the photocatalytic performance [14,15].

This review focuses on the properties of ZnO–GO micro- and nanocomposites, techniques for synthesizing them, and their significant role in the photodegradation and mineralization of organic dyes. Commonly used in industrial processes, dyes—nearly 100,000 of them—are currently present in industrial environments, and every year, approximately 1000 tons of non-degradable ones are released into water resources [16]. In their report, Konstantinou and Albanis, [17] stressed that textile and industrial dyes are the single greatest source of contaminated organic compounds in the world’s bodies of water. This review also critically discusses the properties of ZnO and GO, approaches for synthesizing ZnO–GO nanocomposites, the proper photocatalytic degradation mechanism of dyes, and several factors of dye degradation. 

## 2. ZnO as a Photocatalyst

ZnO is a well-known semiconductor with multiple applications in different fields due to its excellent optoelectronic, mechanical, and electrical properties [13,18]. ZnO exhibits a broad direct bandgap of approximately 3.37 eV, a large excitation binding energy of 60 meV, and deep violet, even borderline ultraviolet (UV) absorption at room temperature. The potentially easy, versatile synthesis of micro- and nanostructured ZnO-based materials via electrodeposition, chemical deposition, the sol-gel process, and hydrothermal methods and their low production costs compared with other semiconductors such as TiO_2_, justify its potential for use in heterogeneous photocatalysis [19,20]. ZnO’s crystalline structure, usually appears in cubic form (i.e., zinc blende), wurtzite, or rock salt structure. The cubic form of ZnO can be stabilized only by escalating the cubic arrangements, whereas the rock salt form of ZnO is rarely formed and, even then, only under extreme pressure [19,20,21,22,23]. The wurtzite structure of ZnO possesses extraordinary thermodynamic stability compared with the other two forms. 

**Figure 1:**
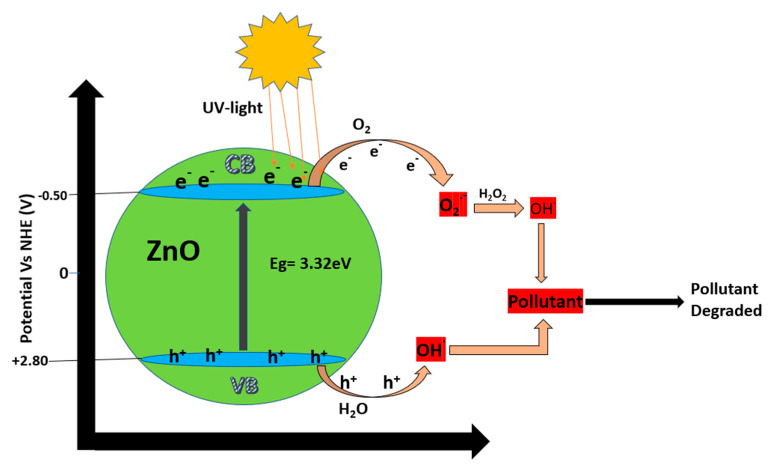
Photocatalytic mechanism of ZnO in wastewater.

When ZnO photocatalysts are irradiated with a photonic energy equal to or greater than the excitation energy (E_g_)—for example, solar light—the electrons from the valence band (VB) are promoted to the conduction band (CB), thereby generating electron-hole pairs (e^−^/h^+^). As shown in Figure 1, those electron-hole pairs can migrate to the surface of the ZnO, where h^+^ reacts with hydroxide ions or water, if not both, to produce hydroxyl radicals, and e^−^ reacts with oxygen to produce superoxide radicals and, in turn, hydroxyl radicals. Forming various ROS such as superoxide radicals and hydroxyl radicals, all of which are powerful oxidizing agents, is critical for wastewater treatment. The ROS formed rapidly react with the adsorbed organic pollutants on the ZnO surface, which results in the formation of intermediate compounds that can be easily mineralized to form nontoxic chemicals such as carbon dioxide, water, and inorganic compounds. The broad direct bandgap, however, restricts the potential use of ZnO under solar light irradiation, because solar light consists of approximately 5% UV light, 43% visible light, and 52% infrared light. As such, only 5% of solar light is available to excite electrons from the VB to the CB, as well as to achieve the rapid recombination of electron–hole pairs, which inhibits the practical use of ZnO. Therefore, extending the light absorption to the visible domain, increasing carrier mobility, and reducing the recombination by separating photogenerated electrons and holes are essential to applications of ZnO [13,19,20,21,22,23,24]. On top of that, ZnO has several deficiencies, including limited flexibility, the aggregation of nanostructures during irradiation, and high photocorrosion, all added to its high recombination and broad direct bandgap. Of those deficiencies, photocorrosion is an important parameter to consider, because photocatalyst leaching and dissolution during irradiation can significantly shorten the lifetime of photocatalysts as well as introduce other pollutants, especially Zn(II) ions, into treated water [25,26,27,28]. 

To date, various strategies have been examined for modulating the ZnO bandgap to minimize the recombination losses of charge carriers, extend the light response to visible light, and improve the photocorrosion resistance. In that regard, different doping techniques (i.e., cationic, anionic, rare earth, or co-doping) and strategies of thin-film deposition, implantation, nanoparticle deposition with noble metals (e.g., Pt, Pd, Au, and Ag), or semiconductor coupling with other metal oxides (e.g., ZnO–TiO_2_ and ZnO–SnO_2_) have been investigated [29,30,31]. The heterojunction of metal oxide, however, can endanger human health due to its increased toxicity and potential cytotoxicity in nanomaterials as a consequence of their nanoscale size and the improved production of radicals and ROS when photocatalysts are irradiated, which facilitate, for example, the penetration of photocatalysts into the human skin. However, the formation of heterostructured composites based on semiconductor nanomaterials not only improves the catalytic activity but may also reduce possible adverse health effects by supporting and dispersing ZnO nanomaterials (i.e., producing larger structures and decreasing their interaction with or penetration into the human body or other organisms) without significantly decreasing the effective surface area [32]. The formation of heterojunctions and nanocomposites with carbon-based nanomaterials such as fullerenes, GO, carbon nanotubes, and graphene has been demonstrated to be an effective strategy for photocatalytic applications driven by visible light [33]. The use of nanocomposites also enhances structural and electronic properties which is possible with simple photocatalysts [34]. 

Another important parameter to be considered for photocatalytic applications is the photocatalyst architecture, which can significantly improve its light trapping ability, capacity to adsorb pollutants, and consequently global photocatalytic performance [13,24,25,26]. In that sense, ZnO offers a versatile means of synthesis that allows its existence in one-dimensional (1D), two- dimensional (2D), and three-dimensional (3D) structures. Among other architectures and shapes, 1D materials (e.g., needles, ribbons like structures, nanorods, wires, springs, combs, and ring like structures), 2D materials (e.g., nano-pellets and nano-sheets), and 3D materials (e.g., flowers, dandelions, and snowflakes) have been fabricated [22].

## 3. Graphene as a Supportive Material for Photocatalysis

**Figure 2:**
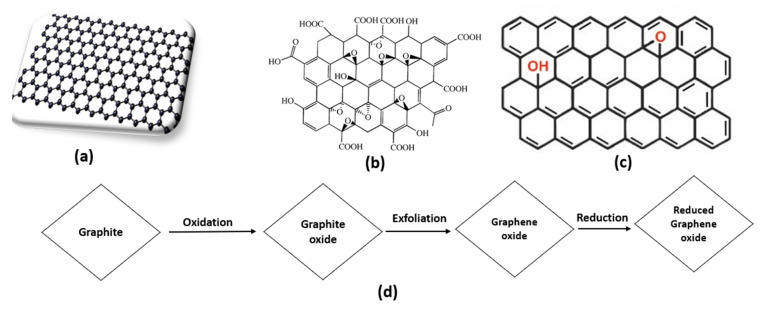
Structure of (**a**) graphene, (**b**) graphene oxide (GO), and (**c**) reduced GO (rGO), along with (**d**) the route of synthesizing rGO by reducing GO.

In general, carbon exists in diamond, graphite, and amorphous forms, which exists depending on carbon’s atomic arrangement and properties. Recently added to the carbon group, graphene contains 2D carbon with an atom-thick nano-sheet. Such a unique compound is closely related to carbon nanotubes and all other nanomaterials, because graphene is one of their basic elements. Graphene has been developed into a leading material in an extensive range of fields and applications, including the conversion and storage of energy [35]. There are various forms of graphene, as illustrated in Figure 2. 

Although these carbon-based nanomaterials are used mostly in fabricating heterojunction with various applications, they also afford remarkable opportunities for developing redox vibrant media and catalysts in water purification owing to their wide surface area and optical, catalytic, and electronic properties, albeit depending upon their shape [36]. Carbon-based nanomaterials also pose excellent adsorption properties due to their exceptional porosity and wide surface area, which provides many active sites that benefit wastewater treatment [37]. 

Formed by a single layer of graphite oxide, GO is a relatively novel compound with wide- ranging applicability. In 1859, Benjamin C. Brodie, a chemist at Oxford University, was the first person to experiment with synthesizing GO, followed by L. Staudenmaier in 1898. Thereafter, Hummers et al. continued exploring the compound by employing graphite with a mixture of potassium chlorate and nitric acid in a fume hood [38,39]. Currently, GO is fabricated by using Hummers and Offeman’s process, albeit with slight modifications, as shown in Figure 2d. First, 100 g of graphite powder is mixed with 50 g of sodium nitrate in sulfuric acid. Second, the mixture is cooled in an ice bath until reaching 0–5 °C in order to ensure the absence of impurities. Third, 300 g of potassium permanganate is added to the cooled mixture under agitation, at which point graphite starts to oxidize; potassium permanganate should be added very carefully and in a small amount so as to maintain a suspension temperature under 20 °C. Fourth, the mixture suspension changes into a brownish-grey paste with the development of a small amount of gas after 20 min of mixing. Fifth, mixing should continue for 30 min, after which deionized water is added slowly into the paste, which produces vigorous effervescence along with a rising temperature of 98 °C. Sixth, the diluted paste, upon turning brown, is retained for 15 min. Seventh, more deionized water is added to increase the dilution, after which H_2_O_2_ is also added in order to remove manganese dioxide. Adding peroxide causes the suspension to turn bright yellow. Eighth, the supernatants, in suspension, are filtered, and the yellowish-brown filter needs to be washed 2–3 times. Ninth, the filtered products are heated at 40 °C to a dried powder form. Ultimately, a single atom-thick sheet of GO is obtained [40,41,42]. 

The exceptional properties of graphene-based nanomaterials—extraordinary strength, outstanding flexibility, a high aspect ratio, high thermal and electrical conductivity, and ease of functionalization and modification—have driven their demand for use in various applications in electronics, catalysis, photocatalysis, sensing, and medicine [41,42]. For example, the high-quality production of graphene derivatives such as GO and rGO, their simple and realistic scaling-up, and the biocompatibility have attracted sustained attention for their applicability in developing graphene–semiconductor nanocomposites with tuned textural and surface chemical properties due to their potential for use in environmental and energy applications. GO and rGO are hybrid carbon nanostructures comprising a mixture of sp^2^ and sp^3^ domains due to the surface functionalization with carboxylic, hydroxyl, and epoxy groups, which offer valuable functionalities when combined with compounds of metal or alloyed materials such as Pd, Au, Pt, Ag, and CoPt, as well as polymer and metal oxides such as ZnO, MnO_2_, Fe_2_O_3_, and TiO_2_, all of which can significantly improve the catalytic or photocatalytic performance [43,44,45]. Thus, graphene-based nanocomposites represent a promising area for research on remediating and decontaminating water systems [46].

## 4. Approaches for Synthesizing GO-Based ZnO Nanocomposites

ZnO–GO nanocomposites have garnered significant attention for their applicability in photocatalysis due to their nano-range size and their excellent optoelectronic properties for extending the operation of ZnO photocatalysts into the visible domain, reducing the recombination process and improving photocorrosion resistance [47,48]. Nanocomposites are based on the integration of ZnO nanoparticles or other nanostructures into GO or rGO, which results in good stabilization and prevents the aggregation of graphene-based layers by virtue of strong van der Waals forces existing among graphene-based sheets. The synthesis of novel, more efficient ZnO–GO nanocomposites is becoming increasingly important in materials science [48,49], and some commonly used methods to that purpose are summarized in what follows.

### 4.1. Sol–Gel Method

**Figure 3:**
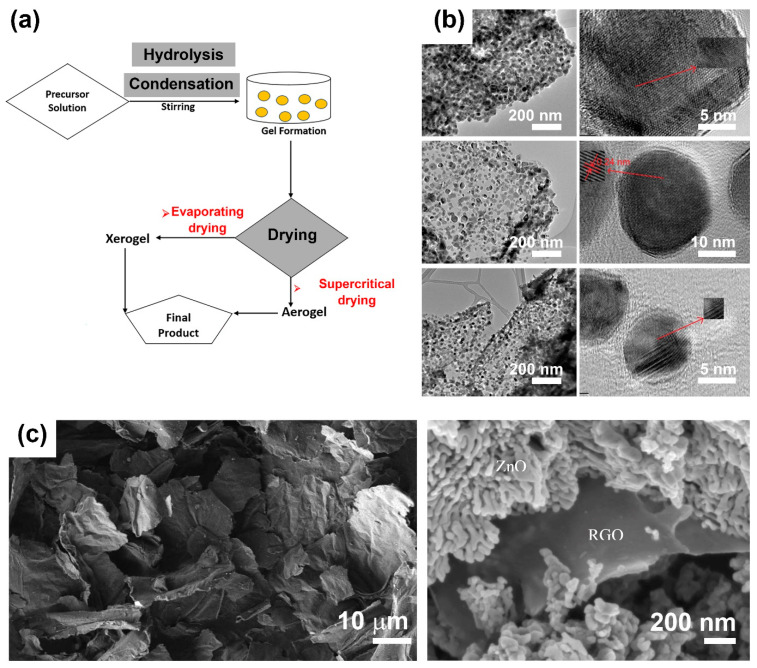
(**a**) Illustration of the sol–gel method, (**b**) transmission electron microscopy images of ZnO–GO nanocomposites (reproduced with permission from ref. [50], with permission from American Institute of Physics, 2014), and (**c**) scanning electron microscopy of rGO sheets (left) and rGO-ZnO core@shell structures (right) (reproduced with permission from ref. [53], with permission from Elsevier, 2020).

First used to fabricate nanocomposites in the late 1980s, the sol–gel method is currently one of the most promising approaches for synthesizing composites and nanocomposites. As shown in Figure 3a, the sol–gel method is a multistep process based on the sequential hydrolysis of metal precursors to produce a metal hydroxide solution, followed by immediate condensation to form a 3D gel. The obtained gel is subjected to a drying process, which prompts the formation of a xerogel or aerogel and, later, the desired product. Aqueous or nonaqueous solvents can be used in the method, and the products exhibit higher porosity and larger surface areas than other nanocomposites. Graphene-based materials, especially GO and rGO, are appropriate precursors to the sol–gel method owing to their high dispersive ability in aqueous and nonaqueous media and functionalities that allow them to react covalently with other compounds. 

In recent decades, the sol–gel method has been used to create ZnO–GO nanocomposites by incorporating ZnO nanoparticles on the surface of GO sheets (Figure 3b) [50,51,52] or the formation of ZnO–rGO core@shell nanostructures (Figure 3c) [53], among others. All of those systems afford a high surface area with enhanced adsorption capabilities and improved photocatalytic performance, which can facilitate the degradation of dyes. 

### 4.2. Hydrothermal and Solvothermal Methods 

**Figure 4:**
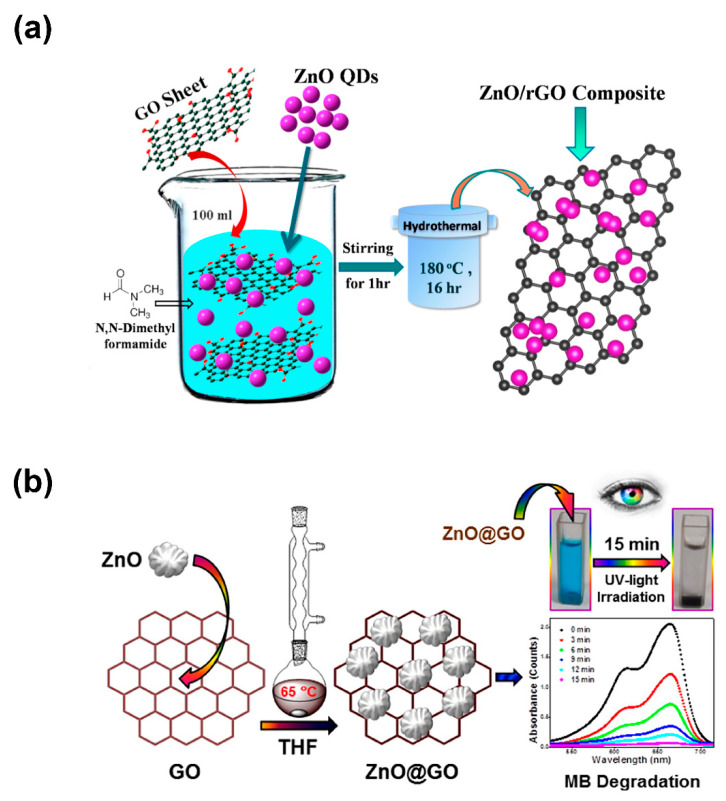
Illustration of the (**a**) hydrothermal and (**b**) solvothermal synthesis of ZnO/GO nanocomposites; reproduced with permission from ref. [60] (with permission from Elsevier, 2016) and [56] (with permission from Springer, 2019), respectively.

In recent decades, hydrothermal and solvothermal methods have also served as powerful means for the efficient synthesis of various nanoscale morphologies (Figure 4), especially for semiconductor materials. Hydrothermal processes involve heterogeneous reactions in aqueous media at temperatures exceeding 100 °C and 1 bar of pressure, whereas solvothermal processes involve replacing the aqueous media with organic solvents. Those processes are performed in closed systems, because the chemical reactions occur with one or various precursors in an aqueous or organic medium at temperatures greater than the boiling point of the corresponding medium. In solvothermal approaches, chemical and physical properties such as dielectric constant, density, polarity, and the interactivity of additives and reactants become especially important in selecting precursors. The hydrothermal or solvothermal synthesis of ZnO–GO or ZnO–rGO is based on incorporating ZnO into graphene-based nanomaterials [54,55,56,57,58]. 

In the hydrothermal method of Marlinda et al. [54] of preparing ZnO–rGO composites, graphene-based nanomaterials produced by Hummers’s method were suspended into deionized water, mixed with a Zn(OH)_2_ solution, basified with sodium hydroxide at 60 °C, and subjected to hydrothermal treatment for 24 h at 180 °C. By contrast, Saravanakumar et al. [55] demonstrated the possibility of fabricating ZnO–GO nanocomposites at a modest temperature range also by using a hydrothermal approach that involved subjecting an ammonia solution containing GO, zinc nitrate, and potassium hydroxide in an air oven for 10 h at 80 and 90 °C. By further contrast, Ahmad et al. [56] reported synthesizing ZnO–Ag–GO nanocomposites by using a simple solvothermal technique involving the use of ethylene glycol as a solvent. As shown in Figure 4b,c, nanocomposites based on GO or rGO can be easily synthesized using a single-step solvothermal method [59,60,61]. 

### 4.3. Direct Growth of ZnO on the Surface of GO

**Figure 5:**
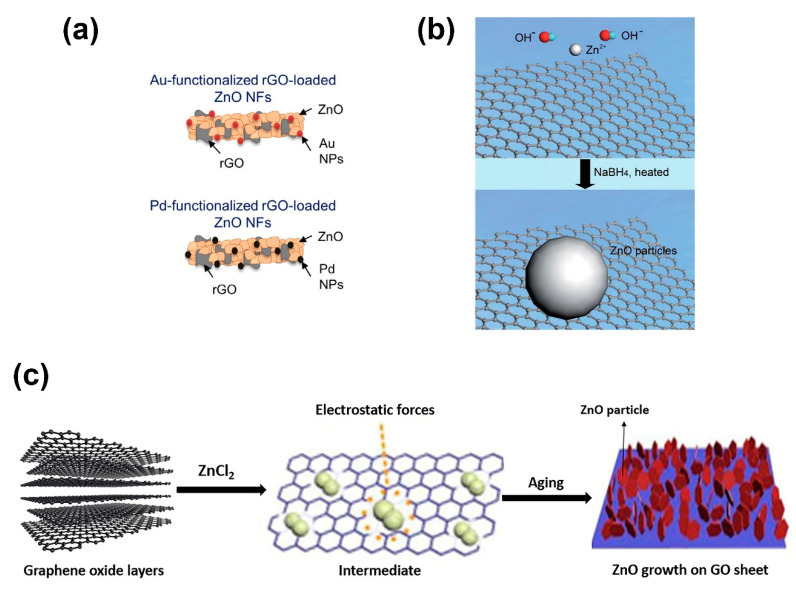
(**a**) Synthesis based on electrospinning for Au- or Pd-functionalized rGO-loaded ZnO nanofibers (reproduced with permission from ref. [66], with permission from Elsevier, 2018), (**b**) illustration of the chemical deposition of ZnO on rGO (reproduced with permission from ref. [62], with permission from RSC Publishing, 2016), and (**c**) illustration of the in situ growth of ZnO on the surface of GO.

Various strategies can be used to directly deposit ZnO on graphene-based materials, including chemical deposition, seed-mediated strategies, electrospinning across electrodes, electrodeposition [62,63,64,65,66]. As shown in Figure 5a, Pt- or Au-functionalized rGO-loaded ZnO nanofibers (NFs) can be easily prepared by combining the sol–gel method or other techniques with electrospinning. The fabrication of ZnO NFs using various ZnO precursor solutions, which can contain GO or rGO to obtain GO-based ZnO NFs, has been amply reported. In the electrospinning process, a high voltage is applied to a polymer droplet precursor placed at the tip of the syringe needle to electrospin the composite NFs. Alternatively, chemical deposition offers a versatile route to deposit ZnO nanoparticles, generally ranging from 10–20 nm in size and with a narrow distribution size, aided by the use of sodium hydroxide and sodium borohydride at 150 °C (Figure 5b). As shown in Figure 5c, ZnO can also be grown on GO or rGO thanks to relatively easy in situ strategy. In that process, zinc (II) ions are adsorbed on the GO or rGO surface and zinc (II) hydroxide precipitates after adding hydroxyl ions, which crystalize in the form of ZnO after thermal annealing. Electrodeposition of ZnO using suspensions of GO and rGO also offers a simple in situ growth method; however, conductive substrates are required for electrodeposition. In general, such methods take advantage of mechanical, chemical, or electrostatic forces that allow the merging of nanocomposite ZnO nanomaterials with graphene-based nanomaterials (Figure 5) [62,63,64,65,66]. 

## 5. Mechanism of the Photocatalytic Degradation of Dyes on GO-Based ZnO Nanocomposites

**Figure 6:**
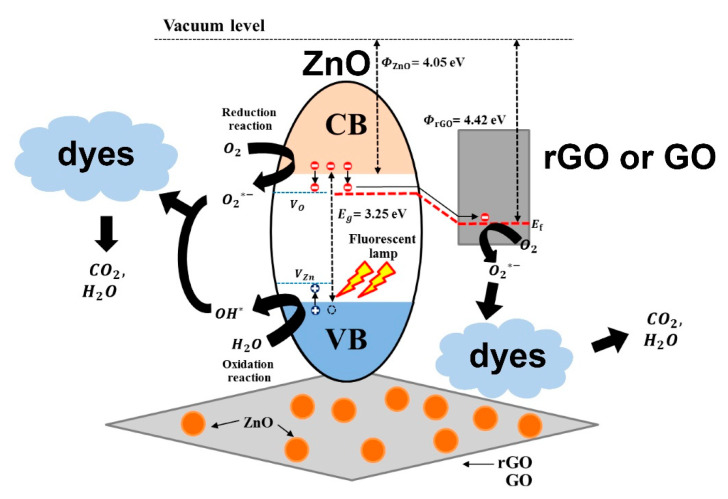
Graphic illustration of the photomineralization of organic dyes by using ZnO–GO or ZnO–rGO nanocomposites as photocatalysts [67].

The key factor in the photomineralization of organic dyes is the formation of ROS such as hydroxyl or superoxide radicals when the photocatalyst is irradiated [13,24]. Various mechanisms can justify the improved photocatalytic performance of GO-based ZnO nanocomposites under visible light irradiation. As implied in the literature, it is plausible for ZnO to support electron–hole pair generation when irradiated with UV light when GO or rGO acts as the carrier pathway. Photocatalytic performance under visible light can be attributed to defect creation and the narrowing of the energy bandgap due to the formation of the ZnO–rGO or ZnO–GO heterostructures. Figure 6 illustrates the photocatalytic mechanism of dye mineralization using GO-based ZnO nanocomposites [60,67,68,69]. Based on that mechanism, when ZnO is irradiated with UV and visible light, the electrons (e^−^) of the VB of ZnO become excited to the CB, thereby leaving holes (h^+^) in the VB, as described in (1).

(1) $$\mathrm{Photons}\;(\mathrm{hv})+\mathrm{semiconductor}\;\to h^{+}\left(\mathrm{VB}\right)+e^{-}\left(\mathrm{CB}\right),$$

The photogenerated electrons, e^−^(CB), can easily transfer to the rGO or GO surface, because the work function of rGO or GO is less than that of the CB of ZnO; consequently, that carrier pathway efficiently enhances the charge separation and prolongs the recombination time for the electron-hole pairs. The photogenerated electrons reacts with oxygen to generate superoxide radicals (2). Next, the superoxide anion reacts with H^+^ to generate first HO_2_• radicals, which react with H^+^ to produce hydrogen peroxide molecules (3). The hydrogen peroxide molecules prompt the formation of additional hydroxyl radicals (4), whereas holes react with water (5) and hydroxide ions (6) to form hydroxyl radicals:

(2) $$e^{\_}+O_{2}\to \;•O_{2}^{-},$$

(3) $$•\;O_{2}^{-}+H^{+}\to HO_{2}•\;\;;\;HO_{2}•+H^{+}+e^{-}\to H_{2}O_{2},$$

(4) $$e^{\_}+H_{2}O_{2}\to \;•OH+OH^{\_},$$

(5) $$h^{+}+H_{2}O\to H^{+}+•\mathrm{OH},$$

(6) $$h^{+}+{\mathrm{OH}}^{\_}\to \;•\mathrm{OH},$$

The photogenerated radicals oxidize organic pollutants such as dyes (7,8) in an oxidation process that continues until organic pollutants are entirely oxidized (i.e. mineralization), which involves the formation of some intermediate that can be difficult to degrade or more toxic than the original organic pollutant. The mechanism of mineralization also depends upon certain experimental conditions such as temperature and photocatalyst loading [60,67,68,69].

(7) $$\mathrm{Organic}\;\mathrm{contaminant}+•\mathrm{OH}\;\to {\mathrm{CO}}_{2}+H_{2}O+\mathrm{other}\;\mathrm{degraded}\;\mathrm{products},$$

(8) $$\mathrm{Organic}\;\mathrm{contaminant}+•\;O_{2}^{-}\;\to {\mathrm{CO}}_{2}+H_{2}O+\mathrm{other}\;\mathrm{degraded}\;\mathrm{products},$$

GO-based ZnO nanocomposites exhibit a high surface area, which can enhance the adsorption of pollutants as well as the formation of ZnO–rGO or ZnO–GO heterostructures. In turn, those processes seem to minimize the recombination losses of charge carriers and extend the light response to visible light.

## 6. Factors Affecting the Photo-Oxidation of Dyes via Photocatalysis

This section discusses several factors that can dramatically and directly affect the photocatalytic performance of GO-based ZnO nanocomposites to degrade, decolorize, and mineralize organic dyes from wastewater. 

### 6.1. Photocatalyst Loading

The effect of photocatalyst loading is especially relevant to photocatalytic behavior. Photodegradation is proportional to the loaded photocatalyst until it achieves an optimized condition due to the high photocatalyst dosage, which increases the global number of active reaction sites, the surface area per unit of volume of reaction media [32,70,71,72], and the amount of photogenerated superoxide and hydroxyl radicals, all of which facilitate the photomineralization of dyes. Conversely, an excessive amount of photocatalyst can hinder photocatalytic performance due to (i) screening effects and the influence of UV light scattering; (ii) photocatalyst agglomeration (i.e., inadequate dispersion and distribution) in unfixed photocatalyst reactors, which reduces the accessible surface area, hinders the absorption of UV and visible light, and obstructs the adsorption of pollutants; and (iii) increased water turbidity, which prevents the dispersion of light into the medium [32,70,71,72]. All of those phenomena suggest that the photocatalyst dosage should be optimized to improve the photocatalytic performance.

### 6.2. Dye Concentration

Several studies have suggested that low or moderate concentrations of dyes improve the kinetics of their photocatalytic mineralization. The surface of photocatalysts can become especially saturated with high concentrations of dyes, because an increased concentration of the target pollutant also increases the kinetics of dye adsorption and competes with the adsorption of other molecules or ions [73,74,75,76]. The rate of photomineralization can be hindered at high concentrations of dyes for two reasons. On the one hand, the active site of photocatalysts can become saturated with dye molecules that compete with the adsorption of water and hydroxide ions and, in turn, reduce the production of ROS such as hydroxyl radicals [73,74,75,76]. On the other hand, the length of the path of photons into the solution is significantly reduced when the concentration of dye increases, meaning that the effective light intensity on the photocatalyst’s surface can also be reduced [73,74,75,76]. 

### 6.3. pH

The pH level significantly impacts the photocatalytic performance in water resources, especially in terms of the catalyst’s surface charge, which significantly modifies the adsorption of dye molecules as well as other ions and molecules [11]. The solution’s pH additionally influences electrostatic interactions on the surfaces of the catalysts, substrates, solvents, dyes, and charged radicals. An important parameter to consider is the point of zero charge (pzc), which indicates the pH at which the net surface charge of photocatalysts equals zero. Consequently, the surface of semiconductors is positive charged when the pH level is less than the pzc and negatively charged when it exceeds the pzc. Therefore, an optimized pH has to be selected that can certify the effective adsorption and photomineralization of organic dyes [74,77,78,79].

### 6.4. Light Intensity

The photogeneration of ROS depends on the absorption of light. Although it is assumed that greater light intensity on the photocatalyst’s surface promotes the kinetics of the formation of superoxide and hydroxyl radicals, it is certain that the photocurrent and, consequently, the electron-hole pair formation are proportional to the square root of the light intensity [11]. The light intensity could also be optimized, because the production of ROS becomes saturated at a concrete value, which makes the reaction rate continuous despite the rising light intensity [74,80,81]. 

### 6.5. Temperature

The photocatalytic degradation depends on the ambient temperature [11]. Although high temperatures can generally improve the rate of photomineralization, the temperature can also affect the kinetics of adsorption. Temperatures greater than the ambient temperature of water could be acceptable at laboratory scale, although not for some technologically relevant applications, including wastewater treatment. At the same time, using high temperatures implies increased energy consumption and higher costs. Altogether, photocatalysts with practical applicability need to be able to function at room temperature [74,82,83]. 

### 6.6. Architecture and Morphology of Photocatalysts 

The efficiency of photodegradation can be enhanced by modifying the surface morphology or architecture of ZnO-based photocatalysts. The use of ZnO-based nanomaterials has attracted considerable attention in the study of photocatalysis due to the high ratio of surface area to volume, added to the already exceptional physiochemical properties and surface functionalities of nanomaterials, which can promote both the adsorption of dyes and their reactivity [12,24,25,26,27,84,85]. ZnO-based nanomaterials can be easily synthesized using various techniques in multiple architectures, shapes and morphologies, including nano-sheets, nano-belts, nanorods, nano- dumbbells, nanowires, nano-spiral disks, neotheropods, and nano-flowers. All of those nanomaterials pose significantly different properties than bulk ZnO. Using nanomaterials is also an excellent strategy to improve the photocatalytic performance of photocatalysts; however, their manipulability, lifetime, reusability, and recyclability need to be improved before they are implementable in technologically relevant applications [12,24,25,26,27].

Biomimetism and bioinspiration can also play important roles in the design of photocatalysts. Because the most efficient architectures in terms of adsorption and light trapping are found in nature, design that mimics natural shapes such as urchins, pollen, and ferns has gained ground in materials science. Bioinspiration in particular has been especially effective in improving light trapping at different angles of incident light as well as the adsorption of pollutants [12,24,25,26,27].

### 6.7. Light Wavelength

The bandgap of semiconductors determines the light wavelength that can be used to produce ROS. The most studied photocatalysts are photosensitive only under the UV domain, which can be divided into UV-A1 (i.e., 315–400 nm), UV-A2 (i.e., 280–315 nm), and UV-A2 (i.e., 100–280 nm). Photocatalysis can benefit wastewater treatment when it involves using solar energy; otherwise, its applicability may be unrealistic. As mentioned, solar light consists of approximately 5% UV light, 43% visible light, and 52% infrared light; for that reason, efforts have been focused on developing photocatalysts driven by visible light in a bid to effectively use sunlight irradiation as an inexhaustible source of energy for wastewater treatment [74].

### 6.8. Effect of Oxidizing Species

Adding oxidizing agents such as potassium peroxydisulfate and hydrogen peroxide to suspensions of ZnO- or TiO_2_-based photocatalysts typically increases the rate of photo-oxidation [80]. Moreover, hydrogen peroxide is presumably multifunctional during photocatalytic degradation, because it can react with superoxide radicals and electrons to generate hydroxyl radicals, which promotes the charge separation and, consequently, the recombination time. A higher concentration of hydrogen peroxide, however, can act as a hole or hydroxyl radical scavenger or else become reactive toward ZnO- or TiO_2_-based photocatalysts, thereby forming peroxy groups that generally hinder the photocatalytic performance [86,87,88]. Hydrogen peroxide has been shown to be more efficient in the photocatalytic dye’s degradation than potassium peroxydisulfate, especially in terms of kinetics and the toxicity of byproducts [89]. 

## 7. State-of-the-Art of GO-Based ZnO Nanocomposites for the Photo-Oxidation of Dyes

In the last two decades, researchers have invested significant effort into developing GO-based ZnO nanocomposites for the photo-oxidation of organic dyes. As described in Section 5, the formation of ZnO–rGO and ZnO–GO heterostructures seems to minimize the recombination losses of charge carriers and to extend the light response to visible light, which together translates into improved photocatalytic performance, especially under visible light irradiation. Table 1 summarizes the primary ZnO–rGO and ZnO–GO nanocomposites photocatalysts reported in literature from 2016 to 2020 for the photo-oxidation of dyes, particularly the method of synthesis, the photocatalyst morphology, and the photocatalytic conditions and performance [90,91,92,93,94,95,96,97,98,99,100,101,102,103,104,105,106,107,108,109,110,111,112,113,114,115]. 

This section briefly presents a few promising GO-based ZnO nanocomposites developed for the photo-oxidation of dyes. 

**Figure 7:**
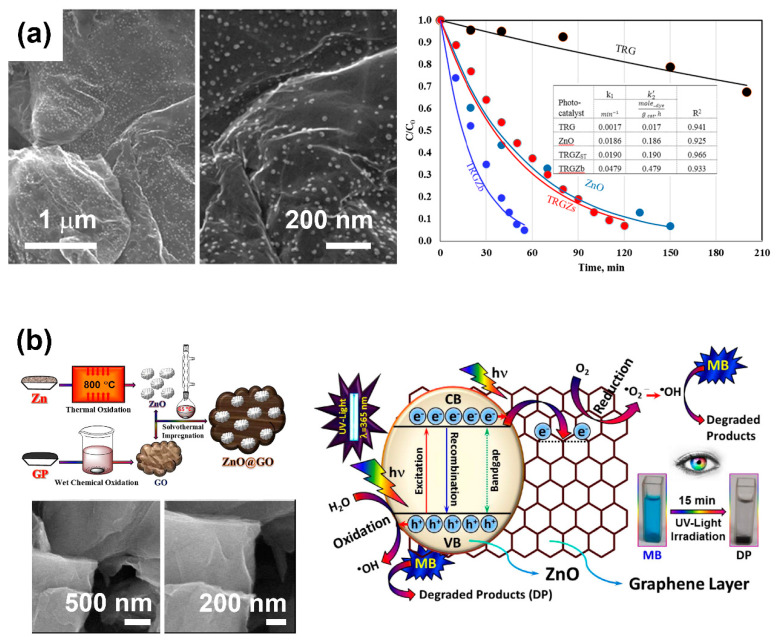
(**a**) Scanning electron microscopy images of ZnO–rGO nanocomposites, showing the uniform distribution of ZnO nanoparticles (left) and the time-dependent, normalized concentration of methylene blue under visible light irradiation for rGO (TRG), ZnO nanoparticles (ZnO) and ZnO–rGO nanocomposites (TRGZ_ST_ and TRGZb) different ZnO loadings (right). Adapted with permission from ref. [90], with permission from Elsevier, 2019. (**b**) Schematic representation of the synthesis and scanning electron microscopy images of ZnO–GO nanocomposites (left) and proposed photocatalytic mechanism for the photodegradation of methylene blue dye under UV-light irradiation using the prepared ZnO–GO nanocomposites. Adapted with permission from ref. [60], with permission from Elsevier, 2016.

Lonkar et al. proposed a simple solvent-free fabrication of ZnO–rGO nanocomposites with superior photocatalytic activity for methylene blue dye photodegradation under visible light irradiation (Figure 7a). In their study, the solvent-free method allowed the uniform incorporation of 9-nm ZnO nanoparticles on GO via the brief ball milling of hydrozincite and GO, followed by thermal annealing [90]. In contrast to multistep solution methods, which produce excessive waste and allow limited production, the solvent-less process offered an effective environmentally friendly strategy. The improved photocatalytic performance of ZnO–rGO nanocomposites was ascribed to the low recombination rate of photogenerated charge carriers and the easy charge transfer induced by the ZnO–rGO heterostructure.

Posa et al. also prepared ZnO–GO nanocomposites by using a simple wet chemical method for the efficient photomineralization of methylene orange dye under sunlight irradiation. The exceptional photocatalytic activity of the nanocomposites was attributed to two factors: the higher charge separation efficiency of electron–hole carriers due to the formation of the ZnO–GO heterostructure, which prolonged the lifetime of charge carriers, and the excitation of the dye, which acted as a visible light sensitizer. The formation of the ZnO–GO nanocomposites resulted in the creation of photocatalysts with highly accessible surfaces (i.e., 158.0 $\;m^{2}g^{-1}$) thanks to the incorporation of GO (i.e., 186.5 $m^{2}g^{-1}$), which possesses a larger surface area than ZnO–GO composites [91].

Atchudan et al. investigated the solvothermal synthesis (Figure 7b) of ZnO–GO nanocomposites based on GO decorated with ZnO nanoparticles. The GO and ZnO nanoparticles were prepared prior to the solvothermal impregnation by using Hummers’s process and thermal oxidation processes, respectively. Those composites were effective in the photodegradation of methylene blue dye under UV light irradiation—to be precise, 98.5% degradation after 15 min of irradiation—due to the improved light adsorption and reduced recombination of electron–holes induced by the formation of a ZnO–GO heterostructure [60].

**Figure 8:**
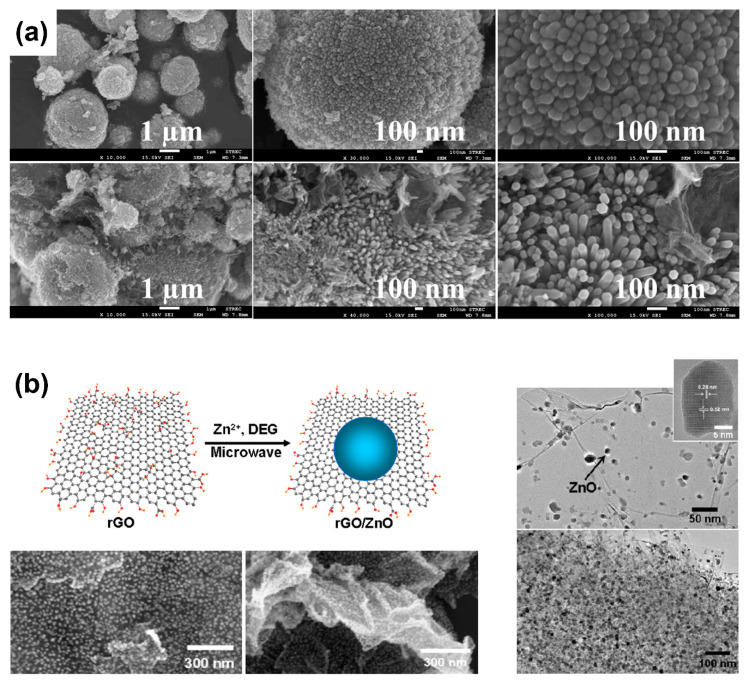
(**a**) Scanning electron microscopy images of ZnO microspheres (top), and ZnO microspheres-rGO composites (bottom). Adapted with permission from ref. [93], with permission from Elsevier, 2017. (**b**) Schematic illustration of the synthesis of rGO/ZnO nanocomposites (left), scanning electron microscopy (left) and transmission electron microscopy (right) images of rGO/ZnO nanocomposites. Adapted with permission from ref. [114], with permission from Elsevier, 2012.

Qin et al. synthesized nanocomposites ZnO microspheres and rGO by using a simple solution method for the photodegradation of methylene blue under UV light irradiation. As shown in Figure 8a, the morphology of ZnO microspheres consisted of ZnO nanorods uniformly distributed with diameters of approximately 30 nm and lengths of approximately 150 nm. The improved photocatalytic performance of ZnO microspheres–rGO nanocomposites, compared to ZnO, was attributed to the reduction of the recombination process of electron-hole charge carriers [93]. 

Using a simple microwave-assisted route in non-aqueous media, Liu et al. fabricated ZnO–rGO nanocomposites based on well-dispersed ZnO nanocrystals on rGO (Figure 8b). The prepared nanocomposites exhibited improved photocatalytic activity in the decolonization of various dyes (i.e., methylene blue and rhodamine B) under visible light irradiation. The authors proposed that the formation of ZnO–rGO significantly reduced the recombination process, which can clearly benefit the photocatalytic performance of the mineralization of dyes. Although the improved photosensitivity of ZnO–rGO nanocomposites in the visible light domain remains controversial, the theory that semiconductors can be sensitized by organic dyes under visible light irradiation can be used to justify excellent photocatalytic degradation under visible light [114]. 

Azarang et al. prepared ZnO–rGO nanocomposites based on ZnO nanoparticles uniformly distributed on rGO by using a simplified sol–gel method, and the synthesized composites were tested for the photodegradation of methylene blue dye under UV irradiation [50]. Similar nanocomposites were fabricated using a hydrothermal approach [106] and chemical deposition [88], which exhibited excellent photocatalytic degradation of azure B and crystal violet dyes, respectively, under UV irradiation. The improved photocatalytic performance relative to ZnO nanoparticles in all of those studies was attributed to the reduction of the recombination process induced by the creation of a ZnO–rGO heterostructure.

This section briefly presented various ZnO–rGO and ZnO–GO nanocomposites recently manufactured for efficiently photodegrading organic dyes such as methylene blue and rhodamine B. The major points of the state-of-the-art synthesis of ZnO–rGO and ZnO–GO nanocomposites are as follows:

- Various approaches to synthesizing ZnO–rGO and ZnO–GO nanocomposites are primarily based on ZnO nano- or microparticles of different sizes incorporated onto the surface of GO or rGO. ZnO nanoparticles and nanorods offer outstanding properties, including large surface areas, which translate into a large number of active sites for pollutant adsorption and photodegradation, high photogeneration of ROS under UV irradiation, high chemical stability, and simple, scalable synthesis. However, high photocorrosion activity and low photosensitivity under visible light irradiation have hindered their potential use. By comparison, GO and rGO have been proposed to be excellent ZnO substrates due to their outstanding physicochemical properties, including extremely high surface areas and their large number of active sites for pollutant adsorption. The formation of ZnO–rGO and ZnO–GO nanocomposites is clearly a potential route for promoting photocatalytic wastewater decontamination. However, the potential of other morphologies or architectures has not been investigated, despite their potential relevance in improving photocatalytic performance. Likewise, the synthesis of photocatalysts has not been explored in relation to posterior integration in fixed or unfixed photocatalytic reactors. Practical applications for photocatalysts need to incorporate efficient synthetic processes that can be easily integrated into the design of reactors. A holistic process that incorporates the synthesis of photocatalysts and reactor design is thus required to better exploit photocatalysis for wastewater treatment.
- The improved photocatalytic activity of ZnO–rGO and ZnO–GO nanocomposites is generally ascribed to the reduction of the recombination losses by the formation of ZnO–rGO or ZnO–GO heterostructures. However, the improved photocatalytic activity under visible light irradiation remains controversial. Photocatalysis driven by visible light is typically justified by the mechanism of semiconductors in the theory of photosensitization. More investigation is thus required to clarify and understand the visible light photodegradation of dyes using ZnO–rGO and ZnO–GO nanocomposites.
- The recyclability and reusability of ZnO–rGO and ZnO–GO nanocomposites have been only superficially explored in the literature.

## 8. Challenges and Perspectives

Extensive efforts have been devoted to the development of various ZnO–rGO and ZnO–GO nanocomposites. To date, studies have shown that the formation of ZnO–rGO and ZnO–GO heterostructures can improve photocatalytic performance by increasing the absorption of light, improving charge separation and transportation, and prolonging functional lifetime. Several studies have also demonstrated the efficient photocatalytic decontamination of water driven by visible light while using several dyes. However, significant challenges have to be addressed in order to overcome the main technical and scientific barriers that prevent their use in technologically and industrially relevant applications:

- In general, the integration of photocatalyst fabrication and reactor design has not yet been realized for wastewater decontamination applications. The potential of heterogeneous photocatalysis relies on the optimal configuration of the reactor, because the photocatalytic performance and stability of photocatalysts heavily depend upon that configuration. Consequently, the design, synthesis, and development of new ZnO–rGO and ZnO–GO nanocomposites to be used as photocatalysts need to incorporate holistic thinking; consequently, future interdisciplinary work between scientists and engineers is required for the success of photocatalysis. That strategy may be relevant to solve one of the chief limitations based on the post-recovery, reusability, and recyclability of ZnO–rGO and ZnO–GO nanocomposites.
- The formation of ZnO–rGO and ZnO–GO heterostructures improved the electron–hole separation, which is critical to improving their global photocatalytic performance. Although considerable progress has been made in synthesizing ZnO–rGO and ZnO–GO nanocomposites, it remains a significant challenge to produce well-defined ZnO–rGO and ZnO–GO heterostructures with controlled size, morphology, and interface. All of those parameters are critical to efficient charge separation. Reducing the recombination phenomenon is insufficient to reduce the cost associated with the UV source capital. Modifying ZnO or ZnO–rGO and ZnO–GO nanocomposites to extend the operation of photocatalysts into the visible domain is another challenge. A fundamental, mechanistic understanding of the photocatalytic degradation of dyes is critical to the synthesis and posterior optimization of the nanocomposites. To understand the charge generation, separation and transportation across those nanoscale interfaces as well as the production of ROS and the role of dyes is critical. To date, investigations have focused only on improving photocatalytic efficiency on the laboratory scale.
- The synthesis of more complex ZnO–rGO and ZnO–GO nanocomposites with well-designed architectures and different shapes or morphologies remains an important challenge. Bioinspiration can be a smart strategy for designing new efficient architectures with improved light-trapping and pollutant-adsorbing capabilities. Despite considerable progress in the synthesis of ZnO–rGO and ZnO–GO nanocomposites, the synthesized nanocomposites are chiefly based on ZnO nanoparticles incorporated onto GO or rGO.
- Stability is another possible challenge in developing photocatalysts due to their short effective lifetimes, owing to both low chemical corrosion and low photocorrosion resistance. In the case of ZnO-based photocatalysts, photocorrosion is more important than chemical corrosion. To date, several studies have focused on improving the photocorrosion resistance of ZnO-based photocatalysts; however, in the case of ZnO–rGO and ZnO–GO nanocomposites, efforts should be intensified. In the field of water decontamination, solving that problem is also relevant, for it is counterproductive to use photocatalysts that release other pollutants (e.g., heavy metals) during the photo-oxidation of organic dyes.
- The fabrication of GO or rGO is also another challenge for graphene-based technology as currently, no scalable and cheaper methods exist to produce large quantities of graphene and its derivatives. It is expected that graphene and its derivatives will find important commercial applications due to their excellent applicability in a wide range of fields. The future of ZnO–rGO and ZnO–GO nanocomposites must also consider the fabrication of GO or rGO.

## Figures and Tables

**Table 1:** Comparison of the synthesis, photocatalytic conditions, and photocatalytic performance of ZnO–rGO and ZnO–graphene oxide (GO) nanocomposites photocatalysts in the photo-oxidation of organic dyes.

Photocatalyst	Synthesis	ZnO Morphology	Dye (Concentration in Ppm)	Photocatalyst Dose (g L^−1^)	Light Source	Removal (%)	Kinetic Constant (min^−1^)	Time of Treatment (min)	Reference
ZnO–GO	Solvothermal	Nanoparticles	Neutral Red (10) Crystal Violet (10) Congo Red (10) Methyl Orange (10)	0.4	UV	~99 ~97 ~68 ~66	3.0 × 10^−1^ 3.3 × 10^−2^ 6.7 × 10^−3^ 6.2 × 10^−3^	20 80 150 150	58
ZnO–GO	Solvothermal	Nanoparticles	Methylene Blue (319)	0.2	UV	98.5	2.5 × 10^−1^	15	60
ZnO–GO	Solvent-free synthesis	Nanoparticles	Methylene Blue (20)	0.75	Visible light	~100	4.8 × 10^−2^	60	90
ZnO–GO	Hydrothermal growth of ZnO nanorod films followed by GO spin coating	Nanorod films	Methylene Blue (3 M)	-	UV	~99	-	450	92
ZnO–GO	Simple solution method	Microspheres	Methylene Blue (10)	1	Visible light	~99	2.1 × 10^−1^	25	93
ZnO–rGO	Hydrolysis and chemical etching approach	Nanorods	Rhodamine B (9.5)	0.05	UV	~92	2.5 × 10^−2^	120	94
ZnO–rGO	Hydrothermal	Nanorods	Methylene Blue (10) Rhodamine B (10) Methyl Orange (10)	0.3	Visible light	~93 ~88 ~75	-	120	95
ZnO–GO	Simple solution method + calcination	Nanoparticles	Safranin T (80)	0.2	Visible light	~100	4.9 × 10^−2^	90	96
ZnO–GO	UV-assisted photocatalytic synthesis	Nanoparticles	Methylene Blue (10)	0.5	UV	~80	1.2 × 10^−2^	120	97
ZnO–GO	Atomic layer deposition	Film	Methyl Orange (13)	0.3	UV	~84	-	270	98
ZnO–GO	Sol-gel	Nanoparticles	Rhodamine B (14)	1.7	Visible light	~99	2.0 × 10^−1^	100	99
ZnO–rGO	Hydrothermal	Nanorod	Direct Red 80 (17.7) Basic Red 80 (20.4)	0.03	UV	~91~83	7.3 × 10^−3^ 7.8 × 10^−3^	186 189	100
ZnO–GO	Ultrasonication + hydrothermal	Nanoparticles	Methylene Blue (20)	0.5	Visible light	~99	1.1 × 10^−2^	120	101
ZnO–rGO	Hydrothermal	Nanorods	Methyl Orange (25)	0.5	UV	~99	5.2 × 10^−2^	60	102
GO–ZnO–GO	Simple solution method	Nanoparticles	O-xylene (40)	0.1	UV	~75	-	45	103
ZnO–GO	Simple solution method	Nanoparticles	Rhodamine B (20)	0.2	UV	~100	9.5 × 10^−2^	65	104
ZnO–rGO	Solvothermal	Nanorods	Orange II (10)	1.2	Solar light	~99	6.6 × 10^−2^	180	105
ZnO–GO	Hydrothermal	Nanoplates	Azure B (5)	0.1	UV	~99	-	20	106
ZnO–GO	Simple solution method	Microspheres	Rhodamine B (5) Methyl Orange (5)	1.0	UV	~99 ~99	6.9 × 10^−2^ 6.2 × 10^−2^	45 45	107
ZnO–rGO	Hydrothermal	Spindle	Methylene Blue (10)	0.05	Visible light	~93	-	180	108
ZnO–rGO	Sol-gel	Lotus	Phenol (940)	0.1	Solar light	~86	1.0 × 10^−1^	20	109
ZnO–rGO	Hydrothermal	Nanosheets	Methylene Blue (40)	0.4	Visible light	~100	-	80	110
ZnO–rGO	Hydrothermal	Nanoparticles	Methylene Blue (1)	1.0	Visible light	~100	1.4 × 10^−2^	60	111
ZnO–rGO	Electrodeposition	Nanowires	Methylene Blue (0.3)	0.025	UV	~23	1.0 × 10^−3^	240	112
